# Recasting the theory of mosquito-borne pathogen transmission dynamics and control

**DOI:** 10.1093/trstmh/tru026

**Published:** 2014-04

**Authors:** David L. Smith, T. Alex Perkins, Robert C. Reiner, Christopher M. Barker, Tianchan Niu, Luis Fernando Chaves, Alicia M. Ellis, Dylan B. George, Arnaud Le Menach, Juliet R. C. Pulliam, Donal Bisanzio, Caroline Buckee, Christinah Chiyaka, Derek A. T. Cummings, Andres J. Garcia, Michelle L. Gatton, Peter W. Gething, David M. Hartley, Geoffrey Johnston, Eili Y. Klein, Edwin Michael, Alun L. Lloyd, David M. Pigott, William K. Reisen, Nick Ruktanonchai, Brajendra K. Singh, Jeremy Stoller, Andrew J. Tatem, Uriel Kitron, H. Charles J. Godfray, Justin M. Cohen, Simon I. Hay, Thomas W. Scott

**Affiliations:** a Department of Epidemiology, Bloomberg School of Public Health, Johns Hopkins University, Baltimore, MD, USA; b Malaria Research Institute, Bloomberg School of Public Health, Johns Hopkins University, Baltimore, MD, USA; c Fogarty International Center, National Institutes of Health, Bethesda, MD, USA; d Center for Disease Dynamics, Economics & Policy, Washington, DC, USA; e Department of Entomology and Nematology, University of California, Davis, CA, USA; f Department of Pathology, Microbiology, and Immunology, School of Veterinary Medicine, University of California, Davis, CA, USA; g Center for Vectorborne Diseases, University of California, Davis, CA, USA; h Division of Integrated Biodefense, Georgetown University Medical Center, Washington, DC, USA; i Institute of Tropical Medicine (NEKKEN), Nagasaki University, Nagasaki, Japan; j Programa de Investigación en Enfermedades Tropicales, Escuela de Medicina Veterinaria, Universidad Nacional, Heredia, Costa Rica; k Department of Defense, Fort Detrick, MD, USA; l Biomedical Advanced Research and Development Authority, Department of Health and Human Services, Washington DC USA; m Clinton Health Access Initiative, Boston, MA, USA; n Emerging Pathogens Institute, University of Florida, Gainesville, FL, USA; o School of Social and Community Medicine, University of Bristol, Bristol, UK; p Department of Biology, University of Florida, Gainesville, FL, USA; q Department of Environmental Sciences, Emory University, Atlanta, GA, USA; r Center for Communicable Disease Dynamics, Department of Epidemiology, Harvard School of Public Health, Boston, MA, USA; s Department of Geography, University of Florida, Gainesville, FL, USA; t Department of Geography and Environment, University of Southampton, Southampton, UK; u School of Public Health & Social Work, Queensland University of Technology, Queensland, Australia; v Spatial Ecology and Epidemiology Group, Department of Zoology, Oxford University, Oxford, UK; w Georgetown University Medical Center, Department of Microbiology and Immunology, Washington, DC, USA; x School of International and Public Affairs, Columbia University, New York, NY, USA; y Department of Microbiology and Immunology, Columbia University College of Physicians and Surgeons, New York, NY, USA; z Center for Advanced Modeling, Department of Emergency Medicine, Johns Hopkins University, Baltimore, MD, USA; aa Department of Biological Sciences, Eck Institute for Global Health, University of Notre Dame, Notre Dame, IN, USA; bb Department of Infectious Disease Epidemiology, Imperial College, London, UK; cc Department of Mathematics and Biomathematics Graduate Program, North Carolina State University, Raleigh, NC, USA; dd Stoller Design Associates, Culver City, CA, USA; ee Senior Graphic Artist, California Science Center, Los Angeles, CA, USA; ff Department of Zoology, Oxford University, Oxford University, Oxford, UK

**Keywords:** Dengue, Filariasis, Malaria, Mosquito-borne pathogen transmission, Vector control, West Nile virus

## Abstract

Mosquito-borne diseases pose some of the greatest challenges in public health, especially in tropical and sub-tropical regions of the world. Efforts to control these diseases have been underpinned by a theoretical framework developed for malaria by Ross and Macdonald, including models, metrics for measuring transmission, and theory of control that identifies key vulnerabilities in the transmission cycle. That framework, especially Macdonald's formula for *R*_0_ and its entomological derivative, vectorial capacity, are now used to study dynamics and design interventions for many mosquito-borne diseases. A systematic review of 388 models published between 1970 and 2010 found that the vast majority adopted the Ross–Macdonald assumption of homogeneous transmission in a well-mixed population. Studies comparing models and data question these assumptions and point to the capacity to model heterogeneous, focal transmission as the most important but relatively unexplored component in current theory. Fine-scale heterogeneity causes transmission dynamics to be nonlinear, and poses problems for modeling, epidemiology and measurement. Novel mathematical approaches show how heterogeneity arises from the biology and the landscape on which the processes of mosquito biting and pathogen transmission unfold. Emerging theory focuses attention on the ecological and social context for mosquito blood feeding, the movement of both hosts and mosquitoes, and the relevant spatial scales for measuring transmission and for modeling dynamics and control.

Mosquito blood feeding and concurrent expectoration creates a wound and a delivery system by which pathogens pass through vertebrate skin to infect vertebrate blood and other target tissues causing diseases such as malaria, dengue, filariasis, Japanese encephalitis, West Nile, Rift Valley fever, and chikungunya. The significant annual health burden of these diseases,^1^ most notably malaria^2–5^ and dengue,^6^ has raised their profile and increased funding for their research and prevention. The recent global financial crisis meanwhile has increased pressure to show a rapid return on this investment.^7^ Donors and government agencies must weigh investments in existing public and veterinary health interventions against the development pipeline for vaccines, drugs, diagnostics, and novel mosquito-control technologies, such as new insecticides and genetic interventions. At the same time, policy makers are asking challenging questions about disease control policies, targets for intervention coverage levels, the costs and benefits of combining various interventions, and the optimal ways to scale up regionally or globally. Given the complex, quantitative nature of control targets and policy for mosquito-borne diseases, dynamic models of mosquito-borne pathogen transmission (MBPT) are indispensable tools for investigating these questions.^8–11^

Mathematical models of MBPT have been used productively to understand and identify key epidemiological features, to measure transmission intensity, and to guide disease control programs.^12,13^ As the need for understanding transmission dynamics and evaluating control options has increased, the types of models being developed and the way they are used have likewise evolved. To understand better the capabilities of current approaches, we recently reviewed the current state of MBPT models.^13^ Here, we extend that review to critique the models, to look at metrics of transmission, and to look at the way those metrics have been combined with models to better inform and more productively shape disease control policies.

## Development of the models and metrics

The basic science and accompanying theory for measuring and modeling MBPT developed slowly from 1877, when Manson showed that mosquitoes transmit filarial worms.^14,15^ Mosquitoes were then implicated in the transmission of malaria in 1897,^16^ yellow fever in 1900^17^ and dengue fever in 1906.^18^ Hundreds of pathogen species are now known to be mosquito-transmitted,^19^ including 38 of clinical significance in humans.^20^ Throughout that history, mathematical models describing MBPT and control catalyzed the development of concepts and metrics that define the study of mosquito-borne pathogens today.^12,13^

The quantitative approach to studying MBPT started with Ronald Ross, who after showing that mosquitoes transmit malaria turned his attention to promoting vector control, and to improving malaria diagnostics. He developed a mathematical theory for vector control through larval source management^21^ and for MBPT,^22,23^ as well as a modeling framework for epidemics in general.^12^ Ross's transmission models and Alfred Lotka's analysis^24^ established solid mathematical foundations for MBPT dynamics.^12^

As Ross contemplated disease control, he recognized the importance of measuring the intensity of malaria transmission. The proportion of the population with a palpably enlarged spleen—the ‘spleen rate’—had been a standard measure of endemic malaria even before Laveran made microscopic diagnosis of malaria possible.^25^ Ross used the prevalence of infection (the proportion of a population found to be infected with malaria parasites by microscopic analysis, called the ‘malaria rate’ or ‘parasite rate’ abbreviated as PR). Driven by a need for more accurate metrics, he developed the ‘thick film’ to improve the sensitivity and specificity of microscopy for diagnosing malaria.^12^ The use of the PR as a metric consequently increased.^25^

Ross also devised mathematical formulas relating the force of infection (FOI), he called it the ‘happenings’ rate to other measurable quantities; i.e., the fraction of a cohort that would be infected over time or at a particular age or in some fixed time period. An important next step came when Muench developed the ‘reversible catalytic’ model into a statistical tool^26^ for both infection prevalence and serology by age as measured by the sero-conversion rate SCR.

Ross's mathematical models describing adult mosquito movement and the spatial scales required for effective larval source management^21^ helped to motivate and justify mark-release-recapture studies to quantify mosquito movement, which was part of operational research during construction of the Panama Canal.^27^ In his books and papers, Ross made the case for developing entomological metrics of the intensity of transmission. In the 1930s, the ‘infective biting density’ was devised^28^ to measure the number of infectious bites, per person, per day or per year; it is now commonly known in malarial studies as the entomological inoculation rate (EIR).^29^ The original pioneering study also compared the EIR to other metrics of transmission: the PR in older children, and the FOI as it was reflected in the pattern of rising age-specific PR from infancy through childhood. The authors noted that although the patterns were roughly consistent with theoretical predictions, epidemiological measures of transmission were obviously much lower than predicted by entomological metrics.^28^

**Figure 1.**
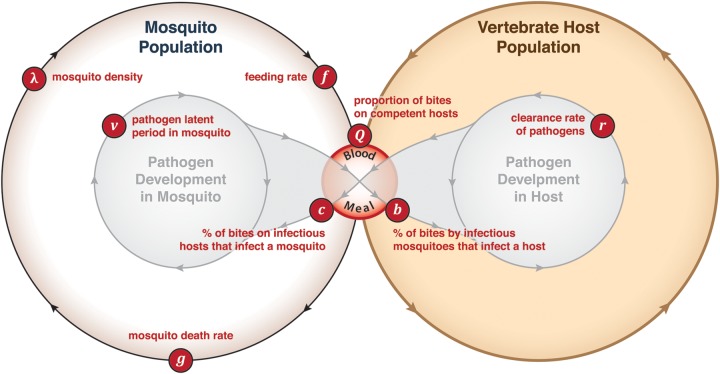
The central and unifying concepts emerging from the Ross–Macdonald model were vectorial capacity and *R*_0_. Vectorial capacity, denoted *v* also called the ‘daily reproductive rate’, describes the intensity of transmission by mosquitoes, the number of infectious bites that would eventually arise from all the mosquitoes that bite a single human on a single day under a set of simplifying assumptions that are both parsimonious and mathematically convenient: the ratio of mosquitoes to vertebrate hosts (*m*) is assumed to be constant; mosquitoes are assumed to feed and die at a constant per-capita rate (*f* and *g*, respectively), to take a constant portion of their bloodmeals on the pathogen's host (*Q*), and to have a constant latent period (*v*). These ‘atomic’ parameters can be combined into three terms that have natural interpretations in the field: the number of mosquitoes biting a person in a day (*mfQ*, for human malaria, which is the human biting rate), the probability a mosquito survives through the latent period (*e*^−gv^), and the expected number of bites on the pathogen's host given by an infectious mosquito (*fQ*/*g*). The product of these quantities is vectorial capacity: *V* = *mf*^2^*Q*^2^e^−*gv*^/*g*. The basic reproductive number, *R*_0_ sums the daily reproductive output of the pathogen, discounted by the inefficient transmission from infectious mosquitoes to susceptible hosts (*b*) or vice versa (*c*), for as long as many days as a host remains infectious (1/*r*): the formula is *R*_0_ = *bVc*/*r*. Note: vectorial capacity assumes *c* = 1. Transmission in the populations of mosquitoes and hosts assumed mass-action kinetics, like two chemical species interacting in a chemostat, so location was vaguely defined, populations are large relative to *R*_0_, biting risk is evenly distributed and redistributed on each blood meal. Most models developed since 1970 continue to adopt most of these assumptions.

In the 1950s, George Macdonald analyzed and synthesized studies from the previous decades describing the epidemiology of malaria and its vectors in a series of landmark papers.^30,31^ His most important achievements are encapsulated in a formula for the basic reproductive number (sometimes called a ratio or rate) for malaria, now called *R*_0_ (Figure 1).^32–34^ Macdonald's formula, which was superficially similar to a threshold criterion developed by Ross, was based on a simple yet compelling mathematical model of the entomological factors associated with transmission, most notably daily mosquito survival (Figure 1). A component of *R*_0_ is the number of infectious bites that would eventually arise from all the mosquitoes that would be infected after biting a single infectious host on a single day, called the daily reproductive number or VC.^35^ VC was also affected by the frequency of mosquito feeding on the pathogen's host, mosquito population density relative to host population density, mosquito survival, and the length of the period during which a mosquito is infected but not yet infectious. The basic reproductive number, *R*_0_, describes the expected number of times a pathogen is transmitted from one host to another after one complete pathogen life cycle (Figure 1). A threshold condition for a pathogen to invade a population is *R*_0_>1, because each infected host would, on average, have to transmit the pathogen to more than one infected host. As a metric of transmission intensity, *R*_0_ thus encapsulates most aspects of the transmission process, and Macdonald proposed it as a threshold condition for pathogen persistence in the absence of control.^33^

Macdonald pioneered a quantitative theory of vector control in an era when contact pesticides (e.g., DDT for indoor residual spraying) were being used extensively for the first time. Macdonald's analysis was based on a mathematical sensitivity analysis of the formula for *R*_0_,^31^ which showed that the potential for transmission was affected by mosquito longevity in two ways: an infected mosquito must survive long enough for the pathogen to mature, and the mosquito must blood feed while infectious, so the longer it lived, the more infectious bites it would deliver. Because the latent period for infections in the mosquito, called the ‘extrinsic incubation period,’ is generally longer than most mosquitoes are expected to live (though the length of this period varies depending on the pathogen–mosquito interaction and the environment), the mosquitoes that are most likely to transmit and propagate the pathogen are those that bit an infectious host when they were young and then survived to be quite old.^31,36^ More importantly, since mortality affected these two aspects of transmission in Macdonald's model, the potential intensity of transmission would be highly sensitive to mosquito survival. Macdonald's analysis has since been used to advocate for prioritizing modes of control that reduce adult mosquito survival.

Macdonald argued that measurement of transmission should become a routine part of the Global Malaria Eradication Programme (GMEP, 1955–1969), and his papers and ideas spawned new research on practical methods for measuring mosquito survival under field conditions, the estimation of *R*_0_, the development of a codified set of methods for estimating the parameters comprising VC, and on tests of Macdonald's theory of control.^12^

By the end of the GMEP, a set of quantities had been identified that were relevant for modeling MBPT dynamics and control along with a set of field metrics and statistical methods for measuring transmission. Transmission could be measured in terms of infection prevalence, exposure to a pathogen either epidemiologically (i.e., through the FOI), serologically (i.e., through the SCR), entomologically (i.e., the EIR), or through the entomological potential (i.e., the VC, which can be measured even in the absence of a pathogens). The models made powerful, specific, and testable predictions about the way these quantities would scale across the spectrum of transmission and likely effects of control, and they set the stage for the study of MBPT through to the present day.

Although the GMEP and a program to eradicate *Aedes* mosquitoes from the New World for yellow fever control were being abandoned, the 1970s were an important transition period in the mathematical study of MBPTs. Important advances came with rigorous applications of the catalytic model to estimate incidence from highly age-stratified PR or serological data,^37,38^ and new methods to estimate malaria incidence from longitudinal data.^39^ The practical issues associated with measuring VC spurred more pragmatic approaches for malaria, and in 1980, the WHO returned to using the EIR as a single, comprehensive measure of transmission intensity.^29^ A new mathematical model was developed for understanding transmission of malaria in highly endemic areas, where immunity was an important feature of the system, and it played a key role in the design and interpretation of a large-scale control trial in Garki, Nigeria.^40^ The model was later applied to a similar transmission setting in Kenya.^41^ Studies published between 1965 and 1980 introduced the first simulation models^42,43^ and explored themes of immunity,^40^ seasonality, spatial dynamics, and heterogeneous mosquito biting and its effects on transmission.^44^ The state of the science at that time is summarized in several reviews.^45–47^

## Modern theory

Research themes introduced during the 1970s have been developed through to the present day. The initial focus on malaria has been expanded to include the broader study of other mosquito-borne pathogens, which are transmitted by vectors with different behaviors and ecologies and which have functionally different transmission dynamics and relations to their hosts. As investment in mosquito-borne pathogen research and interventions has been scaled up, there has been a dramatic increase both in the total number of publications in this field as well as those including theory. At least 388 models that included a mechanistic description of transmission were found in 325 publications between 1970 and 2010^13^; approximately half of these were published after 2005. These models were compared using a detailed, 79-part questionnaire to identify the assumptions they made about a wide range of biological features considered by the models. Despite the growing body of theory, most models published in the last 40 years bear a striking resemblance to the Ross–Macdonald model.^13^ Out of 15 core assumptions in the Ross–Macdonald model, most existing models adopted all but one, two, or three of them, leaving most of the underlying framework unquestioned and intact (a detailed description of our methods and findings can be found elsewhere^13^). Does this conservatism reflect the accuracy and appropriateness of the simplifying assumptions required by Ross–Macdonald models, or has the field become canalized to the exclusion of other approaches?

**Figure 2.**
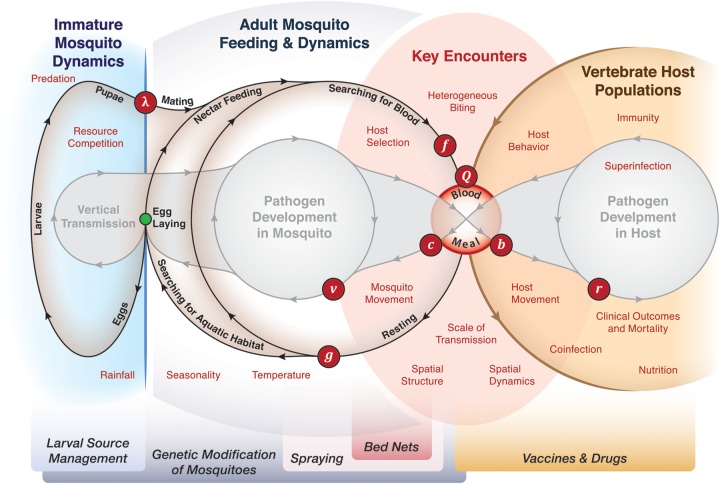
A richer body of theory has been developed since 1970 by elaborating upon the parsimonious assumptions of the Ross–Macdonald model, to include some of the features illustrated here. An important question has been the causes and dynamic consequences of fluctuating mosquito populations over time and space. In some cases, models have coupled adult egg laying with models of aquatic mosquito ecology (blue, at left), including some models that explicitly consider the abiotic and biotic factors that regulate mosquito populations. Other models have considered other aspects of the mosquito feeding cycle, including oviposition behavior and mosquito movement. Other models have expanded on the concepts relating to mixing behavior with models of host selection, heterogeneous biting, or spatial dynamics (red). Some of the greatest differences occur in the expanded models of pathogen infections in hosts, which differ in important ways for malaria, filariasis, and arboviral infections. Despite the rich body of theory that is available, most models continue to adopt the Ross–Macdonald assumptions by default: most models differ from the Ross–Macdonald model in fewer than two key assumptions.

The structure and content of these MBPT models can be understood and classified by the assumptions they make about five distinct components of transmission (Figure 2): pathogen infection dynamics inside the vertebrate host, including immunity; adult mosquito population dynamics and pathogen infection dynamics inside the mosquito; transmission of the pathogen including the mosquito-host encounter and ensuing blood meal from the mosquito to vertebrate host or vice versa, as well as dispersion of the pathogen in infected mosquito or vertebrate hosts; the ecology and population dynamics of immature mosquito population dynamics, involving development from eggs, through four larval instars, pupation and emergence of adults from the aquatic habitats; and egg laying, which links blood feeding adult mosquitoes to immature mosquito populations in both time and space. Not every model of transmission includes every component. Published mechanistic models of pathogen or mosquito population dynamics have generally been developed to address a particular question, so they focus on one or more of these components treating inputs from other components as fixed parameters. A table classifying models by their purpose is also available.^13^

These five components have been extended to address specific biological or control questions involving: various modes of vector control^48–50^; transmission or disease control with drugs or vaccines^51–54^; pathogen evolution and the management of virulence or drug resistance^55^; two or more pathogens and facilitation or competition^54,56^; genetic manipulation of mosquitoes or the evolution of insecticide resistance^57,58^; weather or climate and its relative effects on transmission^59^; impact of parasite burden and aggregation^60,61^; the role of some specific biological mechanism in transmission; spatial or metapopulation dynamics^62^; and multi-host dynamics.^63^

Among the most important innovations in modeling are those that address immuno-epidemiology: models of pathogen population dynamics inside the skin of a vertebrate host, including host immunity and progression from infection to disease.^64–66^ Different mosquito-borne pathogens interact with their human host in very different ways with important consequences for within-host dynamics: for example compare the microparasitic dynamics of chikungunya^67^; interactions among four microparasitic serotypes of dengue^54,68^; the macroparasitic accumulation of filarial worms^60^; and the dynamics of superinfection with genotypically and phenotypically diverse malaria parasites.^69^ Some important consequences of these differences include the relevance of superinfection, the effects of immunity on transmission, and the functional significance of genetic diversity in pathogen populations.

Of great importance for the comparative study of MBPT are functional differences in the immuno-epidemiology of a pathogen-host interaction that constrain the ways transmission can be measured and the sorts of questions that can be addressed for any single disease. Full immunity to filariasis and malaria is not readily developed, and infections persist for long periods of time, so the parasite reservoir in humans is reasonably large. It is thus practical (even if challenging) to measure the prevalence of malaria or filariasis infection in humans and in mosquitoes. Theory suggests that superinfection is an interesting and important metric of transmission for malaria and filariasis, so the study of these parasites has sought methods to measure individual variation in exposure. Because dengue and other arboviral infections cause acute, immunizing infections, the pathogen reservoir is comparatively smaller, and the prevalence of infection in both humans and mosquitoes is much lower. In consequence, individual variation in exposure has received much less attention for arboviral infections, and measures of EIR are more useful for studying malaria, for example, than for dengue. Similar issues affect the comparative ease of studying transmission through the serological status of humans for chikungunya, malaria, dengue, and filariasis. These constraints beg for a comparative approach to MPBT, because even if the vectors differ in some important ways, the observations made from studying pathogen transmission in one system could have great value for understanding the importance of phenomena that could be important but that can't be measured in the others.

A more recent trend that complements modeling studies is the creation, curation, and analysis of databases describing MBPT, including mosquito bionomics, transmission metrics, and other important variables accumulated over more than a century of investigations.^70–73^ Mosquito ecology and MBPT are highly heterogeneous over space and time.^74–77^ At a large scale, it is important to know where transmission is occurring, so maps have played an important historical role in control. The role of maps and the supporting technologies have expanded substantially in recent years with the publication of global maps describing the distribution of malaria^71,78^ and of dengue.^6^ Also of great interest are databases that have aggregated metrics of transmission, especially those studies that have measured two or more metrics at the same time and place, and that investigated the properties of various metrics across space and time or across transmission intensities.^72,73,79^ The marriage of models and large aggregated databases has made it possible to test and apply the models to an extent that has not been possible before.

## Testing theory

Measuring the different components of VC allows the potential intensity of pathogen transmission by any mosquito population to be assessed. But studies adopting this approach have raised important questions about the utility of these: large, poorly quantified errors can arise because of the methods used to catch mosquitoes and estimate bionomic parameters^80^; systematic bias in parameter estimates can arise from fluctuations in mosquito populations^81^ or senescing mosquito populations, or other assumptions of the underlying models; and in making an estimate of VC, errors can be propagated by taking the product of several noisy and potentially biased parameter estimates.^82^

Complementary approaches to VC involve the indirect estimation of *R*_0_ using other field metrics of exposure, based on the assumptions of a mathematical model.^34^ Such methods for malaria include the estimation of the EIR, FOI, or PR. A key observation is that the daily EIR is approximately the product of VC and the net infectiousness of the pathogen reservoir in the vertebrate hosts, i.e., the probability a mosquito becomes infected after feeding on the pathogen's vertebrate host.^12,40^ This makes it possible, at least in theory, to measure VC in two different ways (assuming there is some independent estimate of net infectiousness). The Ross–Macdonald model and most models developed in this tradition assume the FOI is the product of the EIR and the efficiency of transmission per bite, and the relationship between the EIR and the PR is given by simple formulas. These can be tested against the observed values. Other measures include estimating the FOI from changes in serology in a population versus age or time.^83,84^ For dengue and other acute immunizing infections in simple systems, *R*_0_ can be measured by monitoring changes in the number of cases over time.^85^ Measuring changes in the number of cases becomes more difficult for some pathogens that are passed among many mosquito or many vertebrate host species, especially when the epidemiology of the pathogen and presentation of the disease differs for each species. Measuring changes in the number of cases is also difficult for the largely endemic diseases of malaria and filariasis.^34^ Filariasis models focus on the accumulation of worm burdens, and malaria epidemics are restricted to areas with unstable transmission or populations encountering malaria for the first time.

The richness of methods for estimating *R*_0_ provide different ways of cross-validating or ‘testing’ the underlying theory, and unsurprisingly, such studies have also exposed some of the weaknesses due to the simplifying assumptions of the Ross–Macdonald model. Early tests of the theory for malaria that compared estimates of *R*_0_ based on the EIR and FOI, showed large discrepancies because transmission of malaria parasites from mosquitoes to humans was highly inefficient^86^—many infectious bites are required for each infection, which implies a high ratio of EIR to FOI—which is similar to what Macdonald found in his reanalysis of earlier studies.^30^ Similarly, early studies of filariasis independently concluded that transmission is more inefficient than typically assumed.^87^ Further studies of malaria using an aggregated dataset of paired transmission metrics detected a strongly non-linear, empirical relationship that exists between the EIR and the FOI, including ten- to hundred-fold quantitative discrepancies in places with the highest measured transmission.^72^

Published estimates of *R*_0_ for mosquito-borne pathogens are among the highest recorded across all pathogens.^33,34,88^ At first glance, these predictions seem reasonable given the potential for extraordinarily high mosquito population densities and biting rates, but upon more careful examination, and in light of the observed inefficiencies in transmission, they are questionable. Also, the highest estimates are generally based on entomological metrics (i.e., EIR or VC), which are not directly comparable to those collected for directly transmitted diseases. Where non-entomological estimates have been made, which are generally measured using methods that can be compared to estimates made for other pathogens, the estimates obtained are much lower.^34,89^ The extremely high estimates of *R*_0_ obtained from calculations involving VC are due to the implicit assumption that across the spectrum of intensity, the number of infections is proportional to the number of infectious bites.

Heterogeneous biting, a name for the empirical fact that a small fraction of the vertebrate population tends to supply most of the blood meals for mosquitoes, is one factor that could explain what appears to be inefficient transmission because infectious mosquito bites are redistributed in a way that tends to reduce the number of unique individuals who would be infected.^33,72,79,87,90^ Efficiency in transmission also declines if there are only a few vertebrate hosts in the neighborhood who could be infected. Some models of heterogeneous biting have become integrated into the standard Ross–Macdonald model,^33^ but much less work has been done on the spatial scales of transmission and the effects of local mixing between human and mosquito hosts.

## Critiquing theory

Despite the enormous and expanding body of evidence and theory describing MBPT dynamics and control, highly inefficient transmission challenges the applicability of the basic theory. These same questions emerge from attempts to use maps and models together. How heterogeneous is transmission over time and space? What factors give rise to heterogeneous transmission? What are the appropriate scales for modeling MBPT dynamics and control? What are the appropriate sampling frames for measuring transmission?

**Figure 3.**
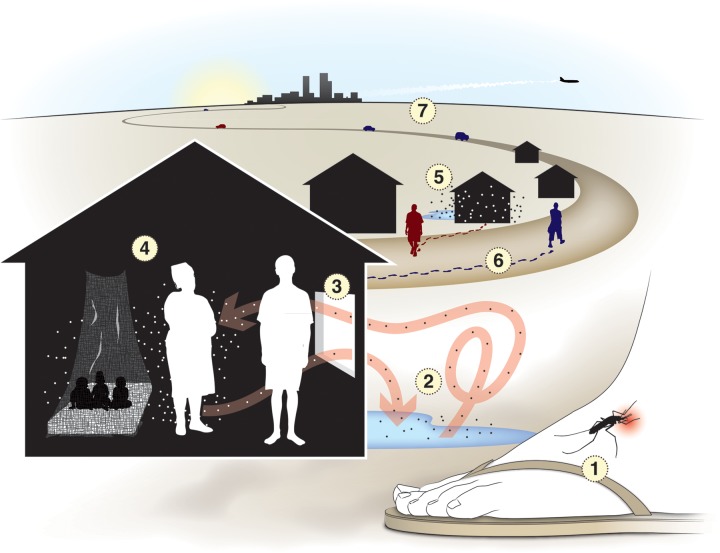
Recasting the theory of transmission requires examination of the factors that give rise to new infections and allow a pathogen to persist. This requires an explicit consideration of the spatial scales that characterize transmission. Existing mathematical theory for transmission of a pathogen by mosquitos focuses on the blood meal itself and factors that affect intensity of transmission (1). New mathematical theory must consider the broader ecological and epidemiological context that determines where and when key encounters between mosquitoes and vertebrate hosts occur. After emerging from aquatic habitats or after laying eggs (2), mosquitoes search for the kinds of habitats where blood feeding typically occurs, such as inside human dwellings (3). Behavioral and physical attributes of mosquitoes and vertebrate hosts, as well as various kinds of vector control strategies (4) determine the outcome of an encounter at mosquito feeding habitats; i.e. a successful blood meal on a particular host, mosquito death or an unsuccessful attempt to feed. Heterogeneity in biting risk among various mosquito blood-feeding habitats depends on mosquito movement (5) and on patterns of human movement (6). The direction of movement among feeding habitats by infected (red) or uninfected (blue) vertebrate hosts (6) and the relative allocation of a host's time at those locations (3, 6) determine the spatial scale of pathogen transmission by hosts. Likewise, alternating mosquito movement between blood-feeding and egg-laying habitats (2) determines the extent of pathogen movement by mosquitoes. At larger spatial scales (7), dispersal of mosquitoes by wind or as cargo and long-distance travel by vertebrate hosts for vacation, business travel, seasonal migration, and other factors determine how the pathogens disperse and persist locally, regionally and globally.

Heterogeneity in transmission is observed at every spatial scale (Figure 3). At small scales (e.g., <100 meters), where mosquito and human behavior and ecology give rise to heterogeneous biting, there are important questions about how mosquito vectors and hosts are distributed across the landscape, how this influences where transmission occurs and how an increased understanding of those processes can be applied to improve efforts to model transmission and apply the lessons to reduce disease. Heterogeneity is also important at spatial scales ranging from kilometers to continents, where ecology and biogeography determine the composition and dynamics of the vector and host communities and the intensity of transmission. An important unanswered question is how the same processes give rise to such a diverse set of patterns across different scales.

The Ross–Macdonald model provides a starting point for dealing with such questions, but it also has limitations. Among the most widely adopted simplifying assumptions of the Ross–Macdonald model was mass-action, a nineteenth century principle from chemistry describing the reaction rates of molecules in an ideal solution. The Ross–Macdonald model assumes that all hosts are identical and equally exposed to pathogens at the same rates, and that the probability of transmission is proportional to the product of host and vector densities. Thus, regardless of the size of the population, there are no epidemiologically important correlations in the distribution of consecutive bites on the same or different hosts. By assuming mass-action it is possible to reduce a great deal of complexity and arrive at a relatively simple expression for *R*_0_.

Macdonald's formula for *R*_0_ is appealing, in part, because it serves several mathematical purposes at once. It is the expected number of secondary infections arising from an initial infection in a non-immune population, and so it gives a deterministic threshold for the pathogen to establish endemic transmission chains. It also provides a single metric of the intensity of transmission that is suitable for comparing the transmission reducing effects of different modes of control, either alone or in combination. The effects of any mode of control on transmission can be compared with the effects of modes of control that reduce adult mosquito population density, which is linearly proportional to *R*_0_. Depending on the patterns of contact, however, the simple scaling relationships that make all these interpretations alike could change because of factors that were omitted from Macdonald's formula.

Pathogen transmission by mosquitoes has been characterized as being highly local and focal, with transmission foci and hotspots.^75^ Hotspots are affected by the juxtaposition of the aquatic habitats suitable for the development of immature mosquito populations to the locations where blood feeding occurs, and by a range of mitigating factors. All transmission involves pathogen movement in either moving infected mosquitoes or moving infected hosts, but what factors determine the size of a focus or the scales that characterize transmission? Ironically, though Ross's first model addressed questions about local mosquito movement,^21^ movement and pathogen dispersal have not become a core part of MBPT theory.

If local processes drive transmission, then the spatial scales that characterize transmission will tend to be small. In simple systems with one host and one vector, effective host population sizes must be small, so that infectious bites are distributed on only a few hosts. In more complex systems, notably zoonotic mosquito-borne pathogens with many vectors and many hosts, transmission patterns are affected by the diversity of less-competent or non-competent hosts.^91^ The more heterogeneous the distribution of bites on those few hosts, the greater the number of bites that would land on the same few hosts, and the lower the expected number of different hosts who would become infected. Because of local mixing and heterogeneous biting, the actual number of new cases arising from an index case is thus strongly limited by the number of hosts that could possibly be bitten. The difference between the number of infectious bites and the number of infections is due to repeated transmission of pathogens to the same few hosts thereby dampening amplification. In more mathematical terms *R*_0_ must be a non-linear function of VC. The functions describing that relationship depend on the distributions of hosts and vectors and the spatial scales that characterize transmission.

Vectorial capacity counts the number of infectious bites arising from a single host on a single day. The formula originally assumed hosts were perfectly infectious, but the formula has also been modified to include vector competence. It does not take into account the redistribution of infectious bites on a finite number of vertebrate hosts in a population with heterogeneous exposure. The problem with inferring transmission by counting infectious bites arising is illustrated by analogy: if *R*_0_ for directly transmitted pathogens were proportional to the number of inocula shed, and by assuming each one of those particles reached and infected a different host, the estimates for other diseases would likely be just as high as for indirectly transmitted mosquito-borne pathogens. What the concept of VC does not account for is the potentially complicated patterns of human-mosquito contact in space and time that distributes infectious bites among a cascade of different hosts with varying infectious status, immune level and innate susceptibility. Just as some inocula are redundant in infecting the same susceptible host many times over, so too are bites by infectious mosquitoes redundant whenever transmission is localized or intense.

Mathematical theory has explored the properties of spatially localized transmission, including the consequences for transmission of heterogeneous biting,^33,44,46,92–95^ local spatial heterogeneity,^93,95^ metapopulation dynamics,^62^ and small population sizes.^33,95,96^ Other frameworks have been developed more recently that show how heterogeneous transmission arises and these lay the foundations for a systematic study of the way these factors vary across systems.^91,97^

Despite highly spatially heterogeneous patterns of transmission, mathematical methods continue to use *R*_0_ as a deterministic threshold for the ability of a pathogen to invade a system, i.e., if *R*_0_>1 then a pathogen will tend to spread. Heterogeneity of all kinds calls into question the value of using a single number to describe how well a pathogen invades. Expressions for *R*_0_, even with heterogeneity, describe how spread would eventually occur, i.e., the asymptotic behavior of the system, without regard to transient phenomena. Such transients are particularly important during invasion if pathogen establishment is stochastic. If the underlying biological determinants of VC are spatially and temporally heterogeneous, then the *expected outcome* will be expected to vary in some way over space and time. The focal nature of transmission raises questions about the relevance of *R*_0_ as a threshold for determining whether the pathogen would tend to invade *here* and *now* even if the threshold has determined that it could invade *somewhere* or *sometime*. Because invasion is a stochastic phenomenon, it matters where and when the pathogen is introduced and what is the *local* VC.^93,95^ To put it another way, it may be possible for a pathogen to invade a potential hotspot, but only if it happens to find it. In this context, it is important to note that there is no mathematical construct for defining a ‘hotspots’ based on dynamical criteria.

## Recasting theory

Development of theory and tests of that theory have raised questions about how actual transmission differs from mass action, and how heterogeneity and poor mixing affect quantitative conclusions about control. Ideas from the Ross–Macdonald model, such as the calculation of thresholds and the sensitivity of transmission to adult mosquito longevity, have been useful. Questions confronting contemporary policy for mosquito-borne pathogens concern quantities describing phenomena that vary through time and space and at different scales.

In order to address these questions we believe new theory should be based on the events that give rise to transmission and accommodate extensive variation in time and space. New models of transmission process should emerge from a quantitative description of the complex local biological interactions among vectors and their hosts. The logic that motivated Macdonald's formula for *R*_0_ is compelling, and it seems likely that any attempt to develop a quantitative index of transmission would adopt many of the same set of parsimonious assumptions. On the other hand, we argue that estimates of *R*_0_ would be more useful if they accounted for the spatial and temporal dimensions of transmission and the way transmission arises from an ecological context and mosquito blood feeding behavior.

An alternative way of understanding the ecology of MBPT, articulated by Hackett for malaria, is to assume that local transmission is a complex puzzle that is, like chess, built up from a few simple pieces.^98^ Following Hackett's logic, Najera et al. proposed an alternative theory of malaria control based on ecological or social contexts giving rise to malaria transmission.^99^ They discussed six specific ecological settings: the African savanna, plains and valleys outside Africa, forest and forest fringe areas, highland fringe and desert fringe, seashore and coastal malaria, and urban malaria. Four specific patterns associated with occupations or social conditions were agricultural colonization of jungle areas, gold and gem mining, migrant agricultural labor, and displaced populations. Macdonald similarly found a categorical approach useful when he proposed three categories of transmission: stable, unstable, and epidemic.^32^ Macdonald was as interested in endemic malaria^32^ as well as epidemics,^100^ but what set his approach apart was the development and application of a quantitative theory based on *R*_0_ to understand both kinds of phenomena. Could the rigor of Macdonald's quantitative approach be applied to codify these categories for malaria, to identify some useful set of categories for mosquito-borne pathogens of humans, or of complex transmission dynamics of pathogens with many mosquito and vertebrate animal hosts? If so, how does transmission in these ecological settings differ in ways that are not captured by *R*_0_ ?

One way to fuse the quantitative methodology of the Ross–Macdonald model with the qualitative view adopted by Hackett and others is to build models that identify the basic components, which will likely include many parts of the formula for VC. What merits more attention is a systematic way of looking at the way complexity arises from the way the pieces fit together. The fundamental questions are about heterogeneity in transmission and the biology that underlies highly local and focal transmission; i.e., poorly mixed populations. Just as the theory of sexually transmitted pathogens successfully recast itself around the concept of heterogeneity in numbers of sexual partners and sexual contact networks in network models, so too must the mathematical theory for mosquito-borne pathogens recast itself around the underlying biology if we are to understand and quantify how ecological and social contexts affect MBPT dynamics and disease control.

A useful concept around which the theory of MBPT can be recast is that of key epidemiological encounters (Figure 3). It is well known that the key encounter for mosquito-borne pathogens is the blood meal, but the spatial context for these encounters has not been carefully examined mathematically. The number, timing, and intensity of encounters are largely a function of how many mosquitoes emerge from aquatic environments located near areas where hosts spend time. The dynamics of larval mosquitoes in aquatic environments are complex and poorly understood, depending on habitat selection by egg-laying adults, biotic and abiotic drivers of developmental success, and how and the extent to which density-dependent mortality operates. Following emergence from these environments, adult female mosquitoes undergo flights for nectar feeding and mating and then an appetitive search to find a blood meal host, a short flight laden with blood to find a place to rest, a search to find a suitable aquatic habitat for egg laying, and then a repeated appetitive quest to find another blood meal host.^101^ Given that the mobility of mosquitoes is on average somewhat limited, locations where blood feeding occurs must be close to other resources such as aquatic habitat and resting sites. Mosquitoes may exercise choice among locations for host seeking and among individual hosts^102^ for blood feeding based on their attributes, including CO_2_ emission, odors,^103^ body size,^104,105^ type of clothing worn, and other factors including elevation, the overall diversity of the vertebrate host community,^91^ and home, nest, or habitat type. It is also important to bear in mind that hosts are also heterogeneously distributed in the environment and are moving targets,^106^ and that hosts can exhibit defensive or avoidance behavior, possibly in response to increased biting by mosquitoes.^107^ The risk of hosts being bitten is a function of where and at what time of day they frequent locations in which mosquitoes are searching for blood meals.

Mosquito biology including the search for egg-laying sites and blood feeding strategies thus emerge as important elements in a new theory that affect transmission as much as blood feeding behavior. Mosquito strategies can range from active questing at night over fairly long distances, such as by *Culex* in agro-ecosystems, to stationary ambush feeding where species such as *Aedes aegypti* or *Aedes albopictus* wait in protected areas until the host arrives. Similarly, the patterns of human activity and mobility in relation to these vector search and feeding strategies are of great importance for understanding transmission. Recent evidence suggests that human social networks are just as important for transmission within cities as mosquito ecology,^108^ and that movement networks are a critical element of transmission within and among countries.^109,110^ Similar problems arise in the study of complex transmission dynamics involving many vectors and many vertebrate hosts where contact networks must contend with the problems of territoriality, seasonal migration, aggregation around resources, and group social structure. In addition to defining the context for key encounters, movement of mosquitoes and hosts at times when mosquitoes are actively feeding jointly govern how pathogens spread during an outbreak and persist over time. There is an urgent need to improve the methods for using data describing mosquito and vertebrate host mobility to understand pathogen transmission dynamics and persistence across scales for pathogens as different as chikungunya, dengue, malaria, and filariasis.

A closely related core concern is that statistical theory must also be developed to inform the spatial scales at which the metrics can be used to estimate transmission in models or to define appropriate sampling frames. The methodology used to analyze transmission metrics has improved substantially since 1970, but like transmission models, there has been very little progress in the basic metrology or in relating those metrics to transmission or control. In particular, the metrics themselves have been poorly validated, and the sampling properties of the metrics (i.e*.*, bias and measurement errors) remain poorly defined.

Concerns about the statistical properties of the metrics are not just hypothetical. The processes of setting coverage targets to meet national goals, of evaluating the impact of mass interventions, of designing trials for interventions that reduce transmission, or of understanding transmission rely on data describing the intensity and scale of transmission. The challenge is that transmission of mosquito-borne pathogens is likely heterogeneous at every scale. In such an environment, what is the appropriate sampling frame for measuring transmission? Having a good metric is often the rate-limiting step for inference, so the practical way forward is to develop theory around the metrics. What windows of space and time are valid for the selected metrics?

If dispersion and the number of hosts in the neighborhood limits transmission, rather than VC, then thresholds on the coverage of vaccines, drugs, and other host-based interventions may not scale linearly with VC. What remains unknown, and is highly relevant for understanding transmission dynamics, is what happens to transmission as locally available hosts become saturated. It may be that, despite the nonlinearities in transmission caused by heterogeneous biting and local transmission, VC-based estimates of *R*_0_ are still relevant in an analysis of vector-based coverage levels and thresholds to eliminate a pathogen from an area. What may also be true is that the thresholds may scale differently for different modes of control depending on the context. What is needed now is a new approach to measuring and modeling these aspects of transmission that can lay the foundations for an improved understanding of MBPT dynamics and control.

## Conclusions

The Ross–Macdonald theory established a critically important framework for the study of infectious diseases, and it has matured substantially over the past century. The central idea is based on the notion of transmission intensity, which is implicit in Macdonald's formula for *R*_0_. There are good reasons to continue to use this approach, while also carefully questioning its many simplifying assumptions. The question is not whether *R*_0_ and accompanying theory is wrong. All models make simplifying assumptions, all scientific inference is based on some kind of model (i.e., including statistical models and all kinds of conceptual models), and simple models are often exceedingly useful. The issue is whether the omission of certain biological features undermines the application of the model. In this case, does including heterogeneous transmission improve conclusions based on *R*_0_ and predictions about the effective control of mosquito-borne diseases?

The observation that most heterogeneity in transmission shares a common spatial dimension begs for the development of a spatially rich theory that can accommodate the limited movement of individual mosquitoes and hosts in variable and sparsely or densely populated landscapes. Movement is especially critical for arboviruses and other strongly immunizing infections where host populations become progressively immune and the number of susceptible hosts can be depleted. Similar issues will likely affect other pathogens, as well. General theory, however, remains tethered to the core assumptions and non-spatial structure of the Ross–Macdonald model.

Analytical insights from theory developed for directly transmitted pathogens may be required to guide the development of detailed simulations, to identify priorities for field research, and ultimately to guide the design of policy. The seeds of the new generation of theory that we call for have been sown by models of mosquito-borne pathogens,^33,44,46,62,91–95,97^ but the continued development, investigation, and widespread adoption of such approaches and connection with the underlying biology have not yet been fully realized. Advances in theory developed for directly transmitted pathogens, including theory describing poor mixing and networks, have not yet been incorporated into the theory for mosquito-borne pathogens. The concepts of networks and social distance have long been ignored, but there is now evidence of their importance for MBPT.^108^ Development of a rich theoretical perspective on networks, motivated by the biology of mosquitoes and their hosts, would be a valuable addition to mosquito-borne pathogen theory.

The success of any new theory will be measured by its utility in specific contexts and by its ability to inform decisions weighing the impacts of various modes of control against their costs. Ross–Macdonald theory provides specific advice about the likely effects of drugs, vaccines, and mosquito control on pathogen transmission, and Macdonald's formula for *R*_0_ is highly compelling and frequently used. On the other hand, it is difficult to place confidence in this kind of advice when tests of the theory continue to expose inadequacies. Should such a theory be used to determine how finite global resources are allocated? For example, should resources be diverted to contain artemisinin-resistant *Plasmodium falciparum* before it spreads beyond Southeast Asia? How should resources be reallocated in light of knowledge of the distribution of pyrethroid-resistant *Anopheles gambiae* in Africa and elsewhere? How could a new vaccine against malaria or dengue be most effectively deployed, and should resources be diverted from existing mosquito control programs to do so? Is pathogen elimination the optimal strategy for a country, and if so, on what time frame? How can limited resources be best used to detect and respond to an introduced exotic pathogen (e.g., Rift Valley fever virus)? Some sort of model will be used to answer all of these questions, but only models that address the unexplored topics identified herein can accurately weigh costs against benefits across different scales of transmission intensity and levels of investment. No single approach is likely to be optimal for every question, so a hierarchy of models and modeling approaches is needed to identify priorities, which will subsequently require empirical validation. Given the inherent uncertainties, the best way to achieve a robust policy recommendation is through the comparison of multiple, independently derived models.

Advancing the theory of mosquito-borne pathogen transmission requires a new synthesis that realistically acknowledges the ecological context of mosquito blood feeding and its quantitative impact on transmission. Specific objectives should be to develop new models that provide guidance about which details are most relevant for increased understanding of transmission dynamics and what types of interdisciplinary collaborations are necessary to make those advancements. These must be rigorously linked to field studies and extensive data on transmission metrics that has already been generated, but there is also a need to develop new theory exploring mosquito ecology and behavior, mosquito and vertebrate host movement, spatial heterogeneity in complex epidemiological landscapes, and the way those factors lead to key epidemiological encounters. These are among the most promising frontiers with potential for high impact in mosquito-borne disease modeling research and its application in disease prevention.
